# The Holographic Principle Comes from Finiteness of the Universe’s Geometry

**DOI:** 10.3390/e26070604

**Published:** 2024-07-17

**Authors:** Arkady Bolotin

**Affiliations:** Department of Public Health, Ben-Gurion University of the Negev, Beersheba 84105, Israel; arkadyv@bgu.ac.il

**Keywords:** holographic principle, finite geometry, entropy, black hole, holographic image, magnification

## Abstract

Discovered as an apparent pattern, a universal relation between geometry and information called the holographic principle has yet to be explained. This relation is unfolded in the present paper. As it is demonstrated there, the origin of the holographic principle lies in the fact that a geometry of physical space has only a finite number of points. Furthermore, it is shown that the puzzlement of the holographic principle can be explained by a magnification of grid cells used to discretize geometrical magnitudes such as areas and volumes into sets of points. To wit, when grid cells of the Planck scale are projected from the surface of the observable universe into its interior, they become enlarged. For that reason, the space inside the observable universe is described by the set of points whose cardinality is equal to the number of points that constitute the universe’s surface.

## 1. Introduction

The holographic principle has arisen in physics as a universal relation between geometry and information both uncontradicted and unexplained by existing theories. The basis of this relation is that a spatial volume *V* with a boundary of area *A* is fully described by no more than $A/4\ell_{P}^{2}$ bits of information, i.e., 1 bit per each 4 Planck areas $\ell_{P}^{2}$ (where $\ell_{P}$ is the Planck scale approximately equal to $1.6\times 10^{-35}\;m$) [1,2,3].

The discovered relation is not trivial. To be sure, discretizing a flat 3-space into an array of primitive cubes of edge length $\ell_{P}$ and assuming that there is a quantum harmonic oscillator in each Planck cube, one can envision a region of volume *V* in this space as a lattice of $V/\ell_{P}^{3}$ oscillators. The lattice has $N^{V/\ell_{P}^{3}}$ states, where *N* is the number of states of each oscillator. Provided N≥2 and *V* is finite, one would expect the number of degrees of freedom contained in the region to grow with *V* in the way that $V/\ell_{P}^{3}\cdot \log N$. However, as it has been proven to hold in a wide range of examples [4], the said number does not exceed the value $A/4\ell_{P}^{2}\cdot \log N$, where *A* is the area of the boundary to the region. This is deeply puzzling. For example, let *V* be the volume of a cube with the edge length $a\gg \ell_{P}$. Then, in accordance with the discovered relation, one would have $a^{3}/\ell_{P}^{3}<3a^{2}/2\ell_{P}^{2}$, i.e., a contradiction: $a<3\ell_{P}/2$.

The universality of the aforementioned relation suggests that it is an imprint of some general law in science. Usually, this law is considered to be due to string theory [5]. Particularly, it was observed that string theory admits a lower-dimensional description in which gravity emerges from it [6]. This observation was the starting point for AdS/CFT correspondence asserting that the boundary of anti-de Sitter space (AdS for short), which is used in string-based theories of quantum gravity, can be regarded as the “spacetime” for a conformal field theory (abbreviated as CFT), which is used to describe elementary particles [7].

As influential as AdS/CFT has proven to be, there is growing skepticism about whether it is adequate to faithfully represent real-world systems. Suffice it to say that the spacetime on which string-based gravitational theories live has more than four dimensions. In that light, AdS/CFT correspondence (or at least the most famous version of it) does not provide a realistic description of gravity. As to the versions of AdS/CFT that may offer a (slightly more) realistic description of gravity, they all imply models of spacetime that are characterized by supersymmetry having no place in our universe [8].

In contrast, the present paper demonstrates that the relation between geometry and information is due to the finiteness of the geometry that describes physical space.

## 2. Physical Space Has a Finite Geometry

There are reasonable grounds to believe that space contains a finite number of points, the corollary being that a geometry of physical space is finite [9]. Let us review those grounds.

In axiomatic set theories, it is imposed that axioms for logic and mathematics must be formulated only on pure or hereditary sets, i.e., ones endowed with no features at all. Contrastively, a vector space—a set with linear operations defined upon it—is not pure: it contains urelements, i.e., objects (vectors) that are not sets but may be elements of sets [10]. So, in accordance with the aforesaid imposition, axioms that are new to or different from ZF, the set of the axioms of Zermelo–Fraenkel set theory, cannot be acquired by interchanging “sets” with “vector spaces” in ZF.

However, on the other hand, there are no mathematical grounds for preferring pure sets to sets containing urelements [11]. What is even more crucial, the metamathematical imposition of pure sets brings about one of the most serious problems in modern physics, viz., the emergence of infinities in well-formed formulas of the classical and quantum formalisms.

Thus, it makes sense to present Hilbert space theory, fundamental to quantum mechanics, in the form of the axiomatic set theory ${\mathrm{ST}}_{\mathrm{Hil}}$ wherein “sets” are replaced with “vector spaces”. As it turned out, all but two axioms of ZF—the axiom of empty set denoted as Empty and the axiom of infinity denoted as Inf—can be translated into the formal language of Hilbert space theory in this manner.

The cause for the exclusion of the axiom Empty is the fact that a set of vectors must include at least the zero vector 0 to be a vector space. Therefore, the notion of empty set is not translatable into the notion of empty vector space. Furthermore, unlike the original axiom Inf stating that the cardinality of a set capable of including some set *X* together with *all* successors of *X* is *infinite*, the translation of Inf into the language of Hilbert space theory asserts that the cardinality of a vector space capable of including some vector space X plus *all* successors of X can be *finite*, in particular 1. Indeed, any vector space must have at least one element, namely, the zero vector space {0}. Since each successor of {0} is ${\left\{0\right\}}^{+}\;:=\;\left\{0\right\}\cup \left\{\left\{0\right\}\right\}=\left\{0\right\}\cup \left\{0\right\}=\left\{0\right\}$, i.e., the zero vector space again, it implies that the vector space {0} including all the successors of itself is the same {0}. With all that, the cardinality of {0} is obviously 1.

In this way, ${\mathrm{ST}}_{\mathrm{Hil}}$ can be considered to be the set of axioms ZF wherein the axioms Empty and Inf have been replaced with their negations, ¬Empty and ¬Inf. In symbols,

(1) $${\mathrm{ST}}_{\mathrm{Hil}}={\mathrm{ZF}}_{\mathrm{fin}}:={\mathrm{ZF}}_{\mathrm{base}}\cup \left\{\neg \mathrm{Empty},\neg \mathrm{Inf}\right\},$$

where ${\mathrm{ZF}}_{\mathrm{base}}$ denotes the “basic” set theory, namely,

(2) $${\mathrm{ZF}}_{\mathrm{base}}:=\mathrm{ZF}\setminus \left\{\mathrm{Empty},\mathrm{Inf}\right\}.$$

While on the subject, let us note down that an ω-model of ${\mathrm{ZF}}_{\mathrm{fin}}$ is a model in which every set has at most finitely many elements (as viewed externally).

Consequently, the formal language of quantum mechanics, L(QM), which has the axioms of Hilbert space theory at its core [12], can be expressed by the formal language of the finite set theory ${\mathrm{ZF}}_{\mathrm{fin}}$ in conjunction with $X_{\mathrm{qsystem}}$, the set of axioms of quantum mechanics that are absent in a set theory. This can be displayed by the following bijection:

(3) $$f\;\;:L\left(\mathrm{QM}\right)\to L\left({\mathrm{ZF}}_{\mathrm{fin}}\cup X_{\mathrm{qsystem}}\right).$$

Consistent with the above mapping, the formal language of classical mechanics, L(CM), must be expressed by the bijection

(4) $$f\;\;:L\left(\mathrm{CM}\right)\to L\left({\mathrm{ZF}}_{\mathrm{fin}}\cup X_{\mathrm{system}}\right),$$

meaning that everything which can be expressed through L(CM) can also be expressed through the collection of axioms of the finite set theory ${\mathrm{ZF}}_{\mathrm{fin}}$ in conjunction with $X_{\mathrm{system}}$, the set of axioms (nonexistent in a set theory) determined by a classical mechanical system being a subject of study (the set $X_{\mathrm{system}}$ is a proper subset of $X_{\mathrm{qsystem}}$, i.e., $X_{\mathrm{system}}⊊X_{\mathrm{qsystem}}$, otherwise classical mechanics cannot be reducible to quantum mechanics).

In line with the reductionist approach [13], the transition from a classical field to a quantum operator field should be analogous to the promotion of a classical harmonic oscillator to a quantum harmonic oscillator. Accordingly, for each field theory FT and its quantum counterpart QFT, the following bijections must hold:

(5) $$f\;\;:L\left(\mathrm{FT}\right)\to L\left({\mathrm{ZF}}_{\mathrm{fin}}\cup X_{\mathrm{field}}\right),$$

(6) $$f\;\;:L\left(\mathrm{QFT}\right)\to L\left({\mathrm{ZF}}_{\mathrm{fin}}\cup X_{\mathrm{qfield}}\right),$$

where $X_{\mathrm{field}}$ and $X_{\mathrm{qfield}}$ denote the sets of axioms that are not present in a set theory; to be specific, $X_{\mathrm{field}}$ comprises axioms that depend on a classical field being studied and $X_{\mathrm{qfield}}$ is the proper superset of $X_{\mathrm{field}}$, i.e., $X_{\mathrm{qfield}}⊋X_{\mathrm{field}}$.

On the condition that any FT and QFT are decidable, every well-formed formula in the formal languages L(FT) and L(QFT) must be consistent with the axiom ¬Inf. This implies that calculations made with FT and QFT are expected to be free from the infinite elements +∞ and −∞.

To check this, let us calculate the zero-point energy $\langle 0|{\hat{H}}_{F}|0\rangle$ of a particle field Hamiltonian ${\hat{H}}_{F}$. This calculation can be viewed as a summation over quantum harmonic oscillators with the zero-point energy ℏω/2 at all points in space:

(7) $$\langle 0|{\hat{H}}_{F}|0\rangle =\frac{\hbar \omega }{2}\sum_{n=1}1.$$

Since the axiom ¬Inf is a part of quantum formalism L(QFT), the existence of an endless sequence of units such as $1,1,1,\ldots ={\left(1\right)}_{n=1}^{\infty }$, where the symbol *∞* denotes an unbounded limit, is not allowed. The implication of this is that the series $\sum_{n=1}1$ must end up convergent. Thence, the vacuum energy ought to be finite. By contrast, if the axiom Inf, not its negation, were to be a part of L(QFT), then the infinite sequence of additions $1+1+1+\cdots =\sum_{n=1}^{\infty }1$ would have the right to be in (7), resulting in the infinite zero-point energy.

Then again, the zero-point energy could always be finite if space were to have a finite number of points, id est, if a geometry of physical space were to be finite.

Before proceeding further, allow us to clarify the difference between finite geometries, bounded (finite) metric geometries, and discrete geometries. For the purpose of the current presentation, one can define a geometry as a system of axioms that identify what “things” are, which constitute “points”, “lines”, “planes”, and so forth.

In terms of this definition, a finite geometry is any of the axiomatic systems that have only a finite number of points.

A finite metric geometry, on the other hand, is an axiomatic system whose set of points is bounded, i.e., all of its points are within a certain (finite) distance of each other [14].

At the same time, a discrete geometry (including the causal set program [15,16,17] whereby spacetime is a collection of points randomly selected in a background continuous space) takes up only objects in which points are isolated from each other in some sense, as for example, the set of natural numbers is a discrete set, i.e., a set of isolated points.

Most important of all, neither a finite metric geometry nor a discrete geometry needs to have a finite number of points.

Unless otherwise stipulated, henceforth, the statements will relate to finite and nonfinite geometries alike.

Let *M* denote a manifold (such as a surface or a space). Assume that the manifold *M* admits a notion of distance between its points; so, *M* is equipped with measures of its regions (i.e., connected parts of *M*) such as area *A* and volume *V*.

Consider a manifold R that is taken to be a region in another manifold *M*, which means that R is deemed to be a subset of *M* having the same dimension as *M* has. For instance, R can be a 3-ball in Euclidean 3-space.

Conceding that the characteristic length L(R) defining the linear scale of the region R is the ratio of the region volume V(R) to the area A(R) of the region boundary, i.e.,

(8) $$L\left(R\right)=\frac{V(R)}{A(R)},$$

and on the condition that ω is given by the expression

(9) $$\omega =\frac{2\pi c}{L(R)},$$

one finds

(10) $$\langle 0|{\hat{H}}_{F}|0\rangle =\frac{\pi \hbar c}{L(R)}\cdot P_{\mathrm{vac}}.$$

where $P_{\mathrm{vac}}$ denotes the series $1+1+\cdots =\sum_{n=1}1$.

## 3. The Holographic Principle

Naturally, the zero-point energy $\langle 0|{\hat{H}}_{F}|0\rangle$ can be presented as the result of multiplying the vacuum energy density $\rho_{\mathrm{vac}}$ by the region volume V(R). So, by allowing $\rho_{\mathrm{vac}}$ to be proportional to the effective cosmological constant $\Lambda_{\mathrm{eff}}$, namely,

(11) $$\rho_{\mathrm{vac}}=\frac{c^{4}}{8\pi G}\cdot \Lambda_{\mathrm{eff}},$$

one can express *the cardinality of the region R* (the number of points constituting R) in terms of $\Lambda_{\mathrm{eff}}$:

(12) $$P_{\mathrm{vac}}=\frac{\Lambda_{\mathrm{eff}}\cdot V\left(R\right)\cdot L\left(R\right)}{8\pi^{2}\ell_{P}^{2}},$$

where $\ell_{P}=\sqrt{\hbar G/c^{3}}$.

At this juncture, let us establish two positive dimensionless ratios:

(13) $$\alpha :=\frac{\Lambda_{\mathrm{eff}}\cdot D_{U}^{2}}{8\pi^{2}},$$

(14) $$\xi \left(R\right):=\frac{L(R)}{D_{U}},$$

in which $D_{U}$ stands for the comoving (“static”) diameter of the observable universe. As $\Lambda_{\mathrm{eff}}$ and $D_{U}$ are both nonnegative constants, the ratio α is expected to remain constant in time. Using α and ξ(R), one can present (12) as the equality

(15) $$P_{\mathrm{vac}}=\rho_{V}^{\;}\left(R\right)\cdot V\left(R\right)=\rho_{\;A}^{\;}\left(R\right)\cdot A\left(R\right),$$

where $\rho_{V}\left(R\right)$ and $\rho_{A}\left(R\right)$ denote the volumetric density and the areal density of the cardinality of the region R, respectively,

(16) $$\rho_{V}^{\;}\left(R\right)=\alpha \cdot \frac{\xi^{2}\left(R\right)}{L\left(R\right)\cdot \ell_{P}^{2}},$$

(17) $$\rho_{\;A}^{\;}\left(R\right)=\alpha \cdot \frac{\xi^{2}\left(R\right)}{\ell_{P}^{2}}.$$

This enables the holographic principle: points that compose a region are entirely contained in the boundary of the region.

## 4. The Entropy in a Manifold

Let C(R) be a system of coordinates, i.e., a set of numbers, that specify the position of each point in a region R. To be qualified as a system of coordinates, C(R) must be such that the operations of addition, subtraction, multiplication, and division are defined and satisfy the closure under addition and subtraction. Considering this, the system of coordinates C(R) must be a field [18].

In a geometry that has only a finite number of points, the cardinality of the field C(R) is a prime power, i.e.,

(18) $$\mathrm{card}\left(C(R)\right)=p^{q},$$

where *p* is a prime number and *q* is the number of points comprising the region R. This means that the system of coordinates C(R) has $p^{q}$ elements, has qlogp degrees of freedom, and can store $q\log_{2}p$ bits of information (that is, $\log_{2}p$ bits per each point in R).

A measure of ignorance about the position of the points in R can be taken to be proportional to $2^{q}$, i.e., the smallest possible size of a coordinate system for a given region. For that reason, the entropy in the region R can be determined as

(19) $$H\left(R\right)=k_{B}\cdot \log_{2}\mathrm{card}\left(C(R)\right)=k_{B}\cdot q,$$

where $k_{B}$ is the Boltzmann constant. The above allows one to construe each point in space as a bit of information.

Recalling that $q=P_{\mathrm{vac}}$ and using the equality (15), one finds that the entropy in a volume V(R) is equivalent to the entropy in the area A(R) of the boundary to R. In symbols,

(20) $$k_{B}\cdot P_{\mathrm{vac}}=H\left(V\left(R\right)\right)=H\left(A\left(R\right)\right),$$

where H(V(R)) and H(A(R)) denote the entropy in V(R) and A(R) such that

(21) $$H\left(V\left(R\right)\right)=k_{B}\cdot \rho_{V}^{\;}V\left(R\right)=k_{B}\cdot \frac{\alpha \xi^{2}\left(R\right)}{L\left(R\right)\cdot \ell_{P}^{2}}\cdot V\left(R\right),$$

(22) $$H\left(A\left(R\right)\right)=k_{B}\cdot \rho_{\;A}^{\;}A\left(R\right)=k_{B}\cdot \frac{\alpha \xi^{2}\left(R\right)}{\ell_{P}^{2}}\cdot A\left(R\right).$$

## 5. The Cosmological Constant Problem

Thanks to the factor $\kappa =8\pi G/c^{4}$, the upper bound on the effective cosmological constant $\Lambda_{\mathrm{eff}}=\kappa \cdot \rho_{\mathrm{vac}}$ laid by observations is interpreted as an observational bound on the vacuum energy density $\rho_{\mathrm{vac}}$. The problem is that the zero-point energy calculated along the lines of the axiom of infinity Inf comes out infinite: recall that the series $P_{\mathrm{vac}}:=1+1+\cdots =\sum_{n=1}1$ diverges under the assumption that Inf holds true. Furthermore, even though the formalism based on Inf can produce (at the quantitative level) finite values of $\rho_{\mathrm{vac}}$ using one or another renormalization scheme, such finite, renormalized values end up being greater than the observational bound by at least 40 orders of magnitude [19,20,21]. This constitutes the conundrum known as “the cosmological constant problem”.

In contrast, within the formalism wherein the axiom ¬Inf is a part, the number of points $P_{\mathrm{vac}}$ constituting space is finite, which, in turn, indicates that the vacuum energy is intrinsically finite.

What is more, on large scales, the space wherein the universe lives is well approximated as three-dimensional and flat [22]. Given that, the manifold $M_{U}$ associated with the universe can be estimated as a three-dimensional Euclidean space. By the same token, the region $R_{U}\subset M_{U}$ representing the space of the observable universe can be looked upon as an ordinary ball (i.e., a 3-ball) of volume $V(R_{U})$ with the boundary of area $A(R_{U})$. Accordingly, $L\left(R_{U}\right)=R_{U}/3$ and $\xi (R_{U})=1/6$.

Hence, considering that (a) a black hole is the most entropic object one can put inside the spherical surface enclosing the region of the observable universe $R_{U}$ and (b) the observable universe is not a black hole, one can find the upper bound of the entropy in the area $A(R_{U})$:

(23) $$H\left(A\left(R_{U}\right)\right)<H_{\mathrm{BH}}\left(A\left(R_{U}\right)\right),$$

where $H_{\mathrm{BH}}$ is the Bekenstein–Hawking entropy [23,24]

(24) $$H_{\mathrm{BH}}\left(A\left(R\right)\right)=k_{B}\cdot \frac{A(R)}{4\ell_{P}^{2}}$$

in which $R=R_{U}$.

As to the entropy $H(A\left(R_{U}\right))$, using (22), it can be evaluated as

(25) $$H\left(A\left(R_{U}\right)\right)=k_{B}\cdot \alpha \cdot \frac{1}{6^{2}\ell_{P}^{2}}\cdot A\left(R_{U}\right).$$

Thus, the bound (23) produces

(26) α<9.

Due to the fact that the ratio α is on par with 1, the effective cosmological constant $\Lambda_{\mathrm{eff}}$ is close to zero. To wit,

(27) $$\Lambda_{\mathrm{eff}}=\frac{8\pi^{2}}{D_{U}^{2}}\cdot \alpha <\frac{18\pi^{2}}{R_{U}^{2}}∼9.3\times 10^{-52}\;m^{-2},$$

where $R_{U}$ is the comoving radius of the observable universe.

The above result exceeds the bound implied by cosmological observations $\Lambda_{\mathrm{eff}}≲10^{-52}\;m^{-2}$ [25] by only one order of magnitude.

## 6. The Volume inside a Black Hole

The relation between geometry and information may help to define a meaningful notion of volume inside a black hole.

For convenience of reference, let us denote the region of a black hole by $R_{\mathrm{BH}}$. Unlike $A(R_{\mathrm{BH}})$, the area of the horizon (i.e., surface) of a black hole remaining the same for all observers, the volume inside a black hole, $V(R_{\mathrm{BH}})$, is not a precisely defined concept: $V(R_{\mathrm{BH}})$ depends on an arbitrary choice of coordinates and as such can be time-dependent or even zero [26,27,28].

This implies that the characteristic length of the black hole’s interior,

(28) $$L\left(R_{\mathrm{BH}}\right)=\frac{V(R_{\mathrm{BH}})}{A(R_{\mathrm{BH}})},$$

is undefined, and so is the ratio $\xi \left(R_{\mathrm{BH}}\right)=L\left(R_{\mathrm{BH}}\right)/2R_{U}$. As a result, one can only argue that the amount of information inside a black hole is contained on the surface of the black hole, namely,

(29) $$H\left(V\left(R_{\mathrm{BH}}\right)\right)=H\left(A\left(R_{\mathrm{BH}}\right)\right).$$

This begs the question: If $V(R_{\mathrm{BH}})$ were to be undefined, how could this expression be true?

However, then, in accordance with (22) the said amount of information can be calculated as

(30) $$H\left(A\left(R_{\mathrm{BH}}\right)\right)=k_{B}\cdot \alpha \cdot \frac{L^{2}\left(R_{\mathrm{BH}}\right)}{4R_{U}^{2}}\cdot \frac{A(R_{\mathrm{BH}})}{\ell_{P}^{2}}.$$

Compare the above with the Bekenstein–Hawking entropy (24) in which $R=R_{\mathrm{BH}}$ gives

(31) $$L\left(R_{\mathrm{BH}}\right)=\frac{R_{U}}{\sqrt{\alpha }}>\frac{R_{U}}{3}.$$

Hence, the Bekenstein–Hawking assumption of black hole entropy is equivalent to the supposition that $L(R_{\mathrm{BH}})$ is one and the same for all black holes and it is commensurate with the comoving radius of the observable universe.

Therefore, the unique interior volume of a black hole (invariable for all observers) can be believed to be

(32) $$V\left(R_{\mathrm{BH}}\right)=\frac{R_{U}\cdot A\left(R_{\mathrm{BH}}\right)}{\sqrt{\alpha }}>\frac{1}{3}R_{U}\cdot A\left(R_{\mathrm{BH}}\right).$$

Owing to the term $h>R_{U}/3$, the volume $V\left(R_{\mathrm{BH}}\right)=h\cdot A\left(R_{\mathrm{BH}}\right)$ is extremely large. By way of example, for a Schwarzschild black hole with the area of the horizon of radius $R_{S}\approx 3\;\mathrm{km}$, $V(R_{\mathrm{BH}})$ is greater than the volume of the sphere in 3-space $R^{3}$ with the radius $r_{\mathrm{sphere}}$, comparable to the distance from the Sun to Mars:

(33) $$r_{\mathrm{sphere}}>\sqrt[{3}]{R_{U}R_{S}^{2}}\approx 226\;\mathrm{million}\;\mathrm{km}.$$

Concerning a geometric shape of the interior region of a black hole, it can be imagined in agreement with the formula (32) as a figure resembling a cylinder with the base of area $A(R_{\mathrm{BH}})$ and the height *h*. Providing the surface area of the hole’s interior coincides with the area of the event horizon, the surface area of the cylinder should be made up of just one component, $A(R_{\mathrm{BH}})$. This means that the said cylinder must be devoid of its side and one of its bases. Needless to say, a geometric shape like this cannot exist in a three-dimensional Euclidean space.

## 7. Magnification of a Holographic Image

The product of $\rho_{V}\left(R\right)$ and V(R) determines the amount of information contained in a region R of volume *V*. Therefore, $\rho_{V}\left(R\right)$ can be considered to be the three-dimensional (volumetric) density of information. Likewise, $\rho_{A}\left(R\right)$ can be seen as the two-dimensional (areal) density of information contained in the boundary to the region R.

Consider $R=R_{U}$. In line with (16) and (17), the degrees of information concentration in $R_{U}$ are

(34) $$\rho_{V}^{\;}\left(R_{U}\right)=\alpha \cdot \frac{1}{12R_{U}\ell_{P}^{2}},$$

(35) $$\rho_{\;A}^{\;}\left(R_{U}\right)=\alpha \cdot \frac{1}{6^{2}\ell_{P}^{2}}.$$

From here it follows that the area containing 1 bit of information is

(36) $$A_{1}=\alpha^{-1}{\left(6\ell_{P}\right)}^{2},$$

and at the same time, the volume composed of 1 bit of information is

(37) $$V_{1}=\frac{1}{3}R_{U}\cdot \alpha^{-1}{\left(6\ell_{P}\right)}^{2}.$$

In a mathematical formalism embracing the axiom ¬Empty, the minimum cardinality of a manifold must be 1. This requires that all *A* and *V* must be bounded from below such that $A\geq A_{1}$ and $V\geq V_{1}$. Provided the sphericalness of *A* and $A_{1}$, as well as *V* and $V_{1}$, it means $r_{\;A}^{\;}\geq r_{\;A_{1}}^{\;}$ and $r_{\;V}^{\;}\geq r_{\;V_{1}}^{\;}$, where

(38) $$r_{\;A_{1}}^{\;}=\frac{3}{\sqrt{\pi \alpha }}\ell_{P}>\frac{1}{\sqrt{\pi }}\ell_{P}\approx \ell_{P},$$

(39) $$r_{\;V_{1}}^{\;}=\sqrt[{3}]{\frac{1}{4\pi \alpha }R_{U}\cdot {\left(6\ell_{P}\right)}^{2}}>\sqrt[{3}]{\frac{1}{\pi }R_{U}\cdot \ell_{P}^{2}}∼3.3\times 10^{-15}\;m\approx \ell_{s}.$$

As it appears, the minimal length scale in a two-dimensional manifold is the Planck length $\ell_{P}$, while the said scale in a three-dimensional manifold is equivalent to the approximate limit of the strong interaction $\ell_{s}∼3.0\times 10^{-15}$ m. That is to say, there are two different minimal length scales in physical space: $\ell_{P}$ and $\ell_{s}$. Such cannot be unless the sphere of radius $r_{\;V_{1}}^{\;}≳\ell_{s}$ is an enlarged holographic image of the circle of radius $r_{\;A_{1}}^{\;}≳\ell_{P}$.

This enlargement explains the puzzlement of the holographic principle. Indeed, suppose, for a moment, that there is no enlargement. Then, covering a surface of the observable universe with primitive circles of radius $r_{\;A_{1}}^{\;}$ would be holographically projected as filling in the interior of the universe with spheres of radius $r_{\;A_{1}}^{\;}$. Since $r_{\;A_{1}}^{\;}\ll R_{U}$, the universe’s interior could be considered as a set of points with a cardinality greatly surpassing the number of points that constitute the universe’s surface:

(40) $$\frac{R_{U}^{3}}{r_{\;A_{1}}^{3}}=\frac{4\pi R_{U}^{2}}{\alpha^{-1}{\left(6\ell_{P}\right)}^{2}}\cdot \frac{R_{U}}{r_{\;A_{1}}^{\;}}\gg \frac{4\pi R_{U}^{2}}{A_{1}}.$$

Obviously, that would be contradictory to the holographic principle. However, due to holographic enlargement, the universe’s interior is discretized by spheres of radius $r_{\;V_{1}}^{\;}$; therefore,

(41) $$\frac{R_{U}^{3}}{r_{\;V_{1}}^{3}}=\frac{4\pi R_{U}^{2}}{\alpha^{-1}{\left(6\ell_{P}\right)}^{2}}\cdot \frac{R_{U}}{R_{U}}=\frac{4\pi R_{U}^{2}}{A_{1}}.$$

Magnification of a holographic image projected from the surface of the observable universe into its interior can be quantified by the ratio

(42) $$\frac{r_{\;V}^{\;}}{r_{\;A}^{\;}}=\sqrt[{3}]{\frac{R_{U}}{r_{\;A}}}.$$

The above means that if the size of the image is $r_{\;V}$, then the “true” size of a projected object $r_{\;A}$ can be estimated as

(43) $$r_{\;A}^{\;}=\sqrt{\frac{r_{\;V}^{3}}{R_{U}}}.$$

For example, the Solar System, whose radius is $r_{\;V}^{\;}∼4.5$ billion kilometers, can be believed to be a holographic image of a two-dimensional structure of size $r_{\;A}^{\;}∼455.1$ kilometers “painted” on the boundary of the observable universe.

By the same token, the “true” distance between the Sun and the nearest known planetary system (Proxima Centauri system) equal to 4.2 light-years when measured through a region of space would be 377.6 million kilometers (about the distance from the Sun to the asteroid belt occupying an orbit between Mars and Jupiter) if it were to be measured on the surface of the observable universe.

In passing, it should be noted that because the weak force has an effective range $\ell_{w}$ which is shorter than $\ell_{s}$ (namely, $\ell_{w}$ is around $10^{-17}$ to $10^{-16}$ m), it can be inferred that the weak interaction takes place only on a surface of the observable universe. Certainly, had $\ell_{w}^{3}$ been a holographic projection of some area on the surface of the observable universe, its “true” scale would have been $∼1.5\times 10^{-39}$ m in accordance with (43), i.e., much less than the Planck length $\ell_{P}$. This may explain why the weak interaction does not produce a bound state, i.e., one in which a particle tends to remain localized in a region of space.

## 8. Concluding Remarks

The following critical comments could be passed on the claims put forward in this paper.

The existence of a minimal length scale modifies the Heisenberg uncertainty principle so that it is impossible to localize a test particle in essence. Subsequently, the notion of “point” breaks down, causing the cardinality of a physical manifold (i.e., the number of points constituting this manifold) to become an ill-defined measure. Due to this, talking about the finiteness of the physical manifold has little sense.

To reply to this criticism, let us first recall that gravity—instead of spoiling the renormalizability of quantum field theories—has long been suggested to lead to an effective cutoff in the ultraviolet, i.e., a minimal length scale $\ell_{0}$ [29,30]. Logically $\ell_{0}$ implies a nonzero minimal uncertainty $\Delta x_{0}$ in position measurements. The latter can be implemented by generalizing the Heisenberg uncertainty principle.

For example, in one dimension, the simplest generalized uncertainty relation incorporates a new term in the right-hand side proportional to ${\left(\Delta p\right)}^{2}$, namely, $\Delta x\Delta p\geq \hbar /2+\beta {\left(\Delta p\right)}^{2}$, where β is a positive parameter independent of Δx and Δp. So, when Δp increases, the new term precludes Δx from becoming arbitrarily small. This results in a nonzero minimal uncertainty $\Delta x_{0}$.

Be that as it may, the existence of $\Delta x_{0}$ does not necessarily mean that there is a minimal length scale $\ell_{0}$ [31]. In fact, the implication $\ell_{0}\to \Delta x_{0}$ is identical to that of $\neg \ell_{0}\vee \Delta x_{0}$. The last reads: “It is true to say that there is $\Delta x_{0}$ but no such thing as $\ell_{0}$”.

Secondly, in accordance with the expression for the entropy in the manifold proposed in this paper (see, for example, Equation (19)), each point constituting the physical manifold equates with a bit of information. Accordingly, the finiteness of the manifold is the notion defined as well as it gets. By way of illustration, the finiteness of the space of the observable universe signifies that the amount of information required to describe this space is limited.

Thirdly, the existence of cutoffs is necessary for treating infinities that arise in calculated quantities of quantum field theories. Whatever such cutoffs are, they all involve nontrivial assumptions like the presence of unknown new physics [32,33]. Meanwhile, another, almost trivial way to avoid infinities in physics in the first place is to negate the axiom of infinity Inf of Zermelo–Fraenkel set theory. This is exactly what has been demonstrated in the present paper.

## Data Availability

The original contributions presented in the study are included in the article, further inquiries can be directed to the corresponding author.
